# Body Composition Analysis of the Clinical Routine Using Air Displacement Plethysmography: Age-Group-Specific Feasibility Analysis among Preterm Infants

**DOI:** 10.3390/nu16162694

**Published:** 2024-08-14

**Authors:** Lennart A. Lücke, Niels Rochow, Katja Knab, Stefan Schäfer, Jasper L. Zimmermann, Anastasia Meis, Stephanie Lohmüller-Weiß, Adel Szakacs-Fusch, Ursula Felderhoff-Müser, Christoph Fusch

**Affiliations:** 1 Department of Anaesthesiology and Intensive Care Medicine, Campus Charité Mitte und Charité Campus Virchow-Klinikum, Charité-Universitätsmedizin, 13353 Berlin, Germany; lennart-alexander.luecke@charite.de; 2 Research Department of Child Nutrition, University Hospital of Pediatrics and Adolescent Medicine, St. Josef-Hospital, Ruhr University Bochum, 44791 Bochum, Germany; 3 Department of Pediatrics, Paracelsus Medical University, Breslauer Str. 201, 90471 Nürnberg, Germanychristoph.fusch@klinikum-nuernberg.de (C.F.); 4 DeuZWEG German Center for Growth, Development and Health Encouragement during Childhood and Youth, 10249 Berlin, Germany; 5 Department of Pediatrics, University Medicine Rostock, 18057 Rostock, Germany; 6 Department of Pediatrics I, Neonatology, Pediatric Intensive Care, and Pediatric Neurology, University Hospital Essen, University of Duisburg-Essen, Hufelandstr. 55, 45147 Essen, Germany; 7 Department of Pediatrics, McMaster University, Hamilton, ON L8S 4L8, Canada

**Keywords:** neonate, air–displacement plethysmography, body composition, lean mass, method analysis, clinical routine, standardization

## Abstract

Body composition assessments using air displacement plethysmography (ADP, PEAPOD^®^) have been introduced into clinical practice at a few neonatal units. To allow accurate body composition assessments in term and preterm infants, a workflow for routine testing is needed. The aim of this study was to analyze the feasibility of weekly routine ADP testing. We analyzed (1) postnatal ages at first ADP assessment, (2) the number of weekly routine in-hospital assessments, and (3) the workload of body composition measurements using ADP in clinical practice on the basis of an retrospective analysis of our own clinical operating procedures. The retrospective analysis of weekly routine ADP testing proved feasible at Nuremberg Children’s Hospital. The analysis of postnatal age at the first ADP test revealed differences across groups, with extremely preterm infants starting at a mean postmenstrual age of 36.6 weeks, very preterm infants starting at 34.2 weeks, and moderate to late preterm infants starting at 35.3 weeks. The mean number of tests before discharge was significantly greater in the extremely preterm group (*n* = 3.0) than in the very preterm (*n* = 2.4) and moderate to late preterm groups (*n* = 1.7). The workload of the procedure is reasonable, at 8–13 min per test cycle. The study proved that weekly routine ADP assessments in preterm infants are feasible. However, the initiation of routine testing in extremely preterm infants starts at a significantly greater postnatal age than in the more mature population. ADP assessments can be safely and easily integrated into clinical practice and may be valuable tools for providing additional information on nutritional status and infant growth. A standardized routine protocol allowing identical measurement conditions across healthcare institutions and a standardized interpretation tool for age-adapted body composition data, however, would improve comparability and usability.

## 1. Introduction

Preterm infants rely on optimum external nutritional management and feeding, whereas a healthy fetus in a healthy pregnancy stays in utero until term and receives adequate placental nutrition for the growth of body mass, organs, and particularly the brain. High survival rates and low neonatal morbidity have led to a focus on improving the quality of survival [1,2]. In preterm infants, an optimum body composition (fat and fat-free mass) is key to lowering the risk for chronic diseases later in life and negative neurodevelopmental outcomes. Both decreased and excessive fat mass increase the risk for metabolic and cardiovascular diseases [3,4,5]. A greater fat-free mass has been related to improved neurodevelopmental outcomes [6,7,8].

Establishing a framework for routine body composition analysis, interpretation, and nutritional intervention is a common goal in neonatal research to further improve the quality of survival [9]. Various methods, such as anthropometric measurements, bioelectrical impedance analysis, dual-energy X-ray absorptiometry, and air displacement plethysmography (ADP), have been used to evaluate body composition [10,11,12].

ADP is a promising, noninvasive, and radiation-free method for body composition analysis in clinical practice. This method has been validated by various studies [13,14,15,16,17] and has been suggested as the gold standard for routine body composition assessments in preterm and term-born infants [12]. An increasing number of centers have introduced regular ADP measurements and shared experiences for routine assessments in neonatal intensive care units (NICUs) [18,19].

These studies, however, have neither reported the feasibility of testing in clinical practice for differing age groups of preterm infants nor established standardized protocols for ADP testing in clinical practice. At the Children’s University Hospital, Nuremberg, body composition measurements were introduced in 2019 into the clinical routine using ADP. To the best of our knowledge, this study is the first to analyze the feasibility of routine ADP assessments in preterm infants.

This study aimed to analyzed the following

- The feasibility of weekly routine ADP assessments for preterm infants;
- Preterm infants’ readiness for first ADP testing across different gestational ages at birth and number of repeated tests during in-hospital routine;
- The workload of body composition measurements using ADP in clinical practice.

## 2. Materials and Methods

### 2.1. Study Design

This quality improvement study was performed from March to September 2021 at the neonatal Level III intensive care unit of the Children’s Hospital at Nuremberg General Hospital, South Campus of Paracelsus Medical School of Nuremberg. The infants were evaluated for testing on the basis of the inclusion criteria (see Section 4.2) and selected from our REDCap NICU database for retrospective analysis [20,21]. Anonymized data were exported and accessed from October 2021 to December 2021.

Prior to the study, the standard operating procedure for ADP measurements was approved by our institutional review board (#SZ_D_028.21-IX-1). In accordance with German professional regulations for physicians, the present retrospective study did not require additional Ethics Committee approval because it was a quality improvement study, with all prior data being available on a routine basis and analyzed anonymously. In our NICU, parents and legal guardians of all our patients are, orally and in writing, informed about our standards of care and routine procedures, their indications, their nature, risks, and benefits. This information is documented by the physician and approved by the parents’ signatures. The use of extended anthropometry by the ADP is included herein.

#### 2.1.1. Nutrition

All infants were fed according to our local clinical guidelines [22]. In general, infants with a birth weight < 1000 g or born at a gestational age < 28 + 0/7 weeks were fed an exclusively human milk diet including a human milk-based fortifier and ready-to-feed milk (Humavant, Prolacta Bioscience Inc., Groot-Bijgaarden, Belgium) for the first 4 weeks after reaching full enteral feeding (150 mL/kg/d). Preterm infants with a gestational age of 28 + 1/7 to 33 + 6/7 weeks received their mother’s own milk (MOM), which was targeted and bovine-fortified (Aptamil FMS, Danone GmbH, Frankfurt, Germany), or preterm formula at 80 kcal/100 mL (Aptamil Prematil, Danone GmbH, Frankfurt, Germany). Breastmilk analysis for target fortification was performed twice per week, and the macronutrient content was adjusted using modules to reach the ESPGHAN recommendations [12,17,18]. Infants born at a gestational age of 34 + 0/7 to 36 + 6/7 weeks received standard fortified MOM (Aptamil FMS, Danone GmbH, Frankfurt, Germany) or preterm formula at 73 kcal/100 mL (Aptamil PDF, Danone GmbH, Frankfurt, Germany). Term-born infants (≥37 + 0/7) were fed MOM or term formula (Aptamil Pronutra Pre, Danone GmbH, Frankfurt, Germany).

#### 2.1.2. Body Composition and Anthropometric Measurements

Body composition assessments were performed via ADP (PEAPOD, COSMED, Inc., Concord, CA, USA), and the details have been previously described [23,24]. ADP relies on a two-compartment model dividing the body into fat mass and fat-free mass on the basis of measured body density data. Body density was calculated from body volume and weight. Body weight was measured via an inbuilt PEAPOD scale. Body volume was calculated as the difference in the compressible air volume of the measuring chamber before and after the subject was placed in the chamber. The estimation of body volume relies on the main assumption that the density of fat mass is constant (0.9007 kg/L) and that the density of fat-free mass is dependent on gestational age. Reference data for fat mass and fat-free mass were obtained by Fomon et al. and Butte et al. [25,26]. Detailed information about the measurement procedure, technical information, and physical conditions of the device has been described elsewhere [23,27].

### 2.2. The Clinical Procedure at the Children’s University Hospital Nuremberg

#### 2.2.1. Inclusion Criteria and Testing Procedure

Weekly body composition measurements were conducted at a predefined time window between 8.30 a.m. and 11.00 a.m. ADP measurements were only performed in clinically stable infants, preferably on Tuesdays. The following inclusion criteria had to be met for eligibility: FiO_2_ of 21%, no respiratory support, and no episodes of significant desaturation (SaO_2_ < 85%) and/or bradycardia (<80/min) requiring stimulation within the last 48 h.

The measurements were performed in the following sequence: (i) body length via a length board, (ii) head circumference via nonstretchable tape, and (iii) body weight and body volume via PEAPOD. The PEAPOD is located in a designated room away from open windows, fans, or heating/cooling ducts to meet the criteria for test location presented in the PEAPOD operator manual (room temperature: 20–28 °C, humidity: 20–70%, pressure: 562–795 mmHg) [27]. The PEAPOD was not moved throughout the study period. The measurements were performed by trained research staff in the PEAPOD room, which was heated to a constant temperature of 26 °C. Body weight was measured to a resolution of 0.1 g via a digital scale integrated within the PEAPOD. The resolution of the length board (Infantometer Seca 416, Hamburg, Germany) and nonstretchable tape was 1 mm. The time required for a single PEAPOD measurement (time within the test chamber) is less than 3 min.

#### 2.2.2. Testing Workflow

The ADP testing routine was developed on the basis of the operator’s manual provided by the manufacturer of the PEAPOD and the previous experience of the research group with the PEAPOD at McMaster University, Hamilton, ON, Canada [28]. PEAPOD measurements were performed by at least two operators: one assigned to operate the PEAPOD (PEAPOD operating nurse) and one assigned to childcare (PEAPOD nurse). The following testing workflow was implemented:

1. Screening: One day prior to the test day, the study nurse screened all neonates in the units. On the test day, eligibility for testing was evaluated using inclusion and exclusion criteria (see Testing Procedure). The attending physician confirmed clinical stability. A list of all infants to be tested on that day was provided to the unit to inform the bedside nurses which infants were being measured.
2. Preparation: The PEAPOD operating nurse was switched on the PEAPOD at least two hours before the first body composition assessment on the day to allow for system warm-up and equilibration. When tests started early in the day, the PEAPOD system was switched on the night before the test day. Automated volume calibration was started before each volume measurement. Manual system calibration was performed at the beginning of each test day. The results from the quality control tests were reviewed once a month.
3. Testing: The PEAPOD nurse transferred infants from the unit to the PEAPOD room after a final infant stability check-up was requested from the nurse at the unit. The infants were undressed prior to testing. Head circumference and length measurements were performed together by the PEAPOD operating nurse and the PEAPOD nurse. The PEAPOD measurements were coordinated and performed by the PEAPOD operating nurse. The detailed instructions for operating the PEAPOD device are described in the manual of the PEAPOD operator [27].
4. Body composition data: The PEAPOD operating nurse was responsible for obtaining and printing the body composition data. The results were visualized on individual body composition graphs and added to the patient’s folder, which was accessible to the physicians, thus allowing interpretation of body composition data.
5. Responsible physicians evaluated body composition tests: However, no standardized recommendations for individual interventions based on body composition results have been published.

### 2.3. Data Analysis

Descriptive statistical analysis was performed. The subjects were grouped by GA at birth into “extremely preterm” (<28 weeks), “very preterm” (28–31 + 6/7 weeks), “moderate- and late preterm” (32–36 + 6/7 weeks), and “term” infants (≥37 weeks) [29]. Time stamps from the PEAPOD database were used to assess the duration per test. For statistical analysis, Microsoft Excel Professional Plus 2016, Microsoft Excel^®^ Office 365 (Redmond, DC, USA) and IBM SPSS Statistics for Windows, Version 26.0 (Armonk, NY, USA) were used. Violin and boxplots were generated via GraphPad Prism version 6 for Windows (GraphPad Software, San Diego, CA, USA; www.graphpad).

## 3. Results

### 3.1. Feasility and Infants’ Readiness

A total of *n* = 429 tests were performed for 260 subjects (*n* = 185 preterm and *n* = 85 term infants; Table 1). Over the course of the study period, a total of 206 preterm infants were born or admitted to our NICU, resulting in 89.8% of the infants being tested following routine clinical protocols. No adverse effects, such as infection, episodes of apnea or desaturation, were observed during the study period.

### 3.2. Weekly Routine Testing

**Figure 1:**
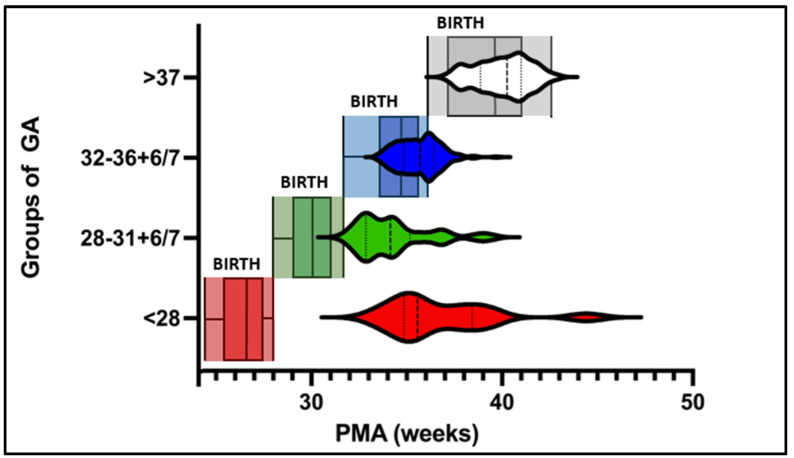
Distribution of gestational age at birth (GA, boxplot) and postmenstrual age (PMA; weeks, Violin plot) at first body composition measurement.

Postnatal age at first ADP testing significantly differed between the groups. Measurements in the extremely preterm group could be initiated at 10.5 ± 3.2 weeks of life. This chronological age was significantly greater than that of the other three groups (very preterm: 4.4 ± 2.4 weeks, moderate and late preterm: 1 ± 0.7 weeks, and term infants: 0.7 ± 0.6 weeks, *p* < 0.01). The mean PMA at the first ADP test was highest in the extremely preterm group (36.6 ± 2.7 weeks). The mean PMAs of the very preterm and moderate-to-late preterm infants were 34.2 ± 1.9 and 35.3 ± 1.1 weeks, respectively (Table 1). The median PMA at the first test in the extremely preterm group was comparable to the median of the moderate–late preterm group (36 weeks). The median PMA at the first test in the very preterm group was comparable to that in the 1st quartile in the moderate–late preterm group (34 weeks, Figure 1). The mean number of tests before discharge was greater in the extremely preterm group (*n* = 3.1 ± 1.4) than in the very preterm (*n* = 2.4 ± 1.4), moderate-to-late preterm (*n* = 1.7 ± 1.1), and term groups (*n* = 1.1 ± 0.2; sig. *p* < 0.01; Table 1).

### 3.3. Personnel Requirements

The time per test was dependent on the number of staff available. When tests were performed by two operators (one PEAPOD nurse and one PEAPOD operating nurse), the time per full testing cycle was 13 ± 3 min. When body composition testing was performed by three operators (two PEAPOD nurses and one PEAPOD operating nurse), the required time per full testing cycle significantly decreased to 8 ± 0.6 min. The test frequency significantly increased due to the greater efficiency of transport between the neonatal unit and the PEAPOD room, as did the undressing and measuring of the subsequent child, with the actual ADP measurement still running.

Throughout the study period, a mean of twelve body composition assessments were performed on the weekly testing day. This corresponds to a weekly mean cumulative work time of 312 min with two operators (12.8 min × 12 tests × 2 operators) and 288 min with three operators (8 min × 12 tests × 3 operators).

## 4. Discussion

The retrospective analysis of the feasibility of routine ADP testing in preterm infants revealed positive results, with almost 90% of infants born at the Nuremberg NICU being tested during the study period. No adverse effects from routine testing were observed. The data revealed a later start of testing in extremely premature infants than in older infants. Longer hospitalizations of premature extremely premature infants resulted in a significantly greater number of repeated body composition tests. The required workload per routine ADP test was reasonable at 8–13 min, depending on the number of nurses available.

### 4.1. Feasibility of ADP Testing in Routine Clinical Practice

Our retrospective analysis demonstrated the potential of ADP to be successfully transferred from a research-only method to a standard clinical method and proposed that almost all clinically stable infants in the NICU can be successfully tested without adverse effects. While Bell et al. recommended using body composition assessment as a tool for routine clinical practice, Aljanini et al., similar to us, demonstrated the feasibility of introducing ADP into routine clinical practice [19].

The data indicate that during the study period, 89.8% of preterm infants admitted or born at our NICU underwent routine ADP assessments. A small fraction of the remaining 10.2% of infants who could not be assessed in a clinical setting were affected for three main reasons: (1) clinical instability, (2) nursing during scheduled assessment, and (3) early discharge or transfer to a different hospital.

### 4.2. Routine Testing

#### 4.2.1. Postnatal Age at the First Test

Different institutions have recently implemented ADP assessments in clinical practice [18,19,24]. Although the clinical settings and assessment procedures were methodically described, they were neither retrospectively analyzed nor formulated as standardized protocols for routine assessment.

In our cohort, body composition measurements were initiated significantly later at a higher postmenstrual age in infants with a lower GA. This phenomenon is most likely due to the periods of clinical instability and prolonged respiratory support in the groups born extremely or very prematurely. Bruckner et al. reported similar findings. The first PEAPOD test was initiated at significantly greater postnatal ages in extremely preterm infants (days of life: 89 ± 28) than in very preterm infants (days of life: 39 ± 15) [30].

Our sample size (*n* = 429) is large enough to describe the feasibility of ADP assessments for different groups of premature infants. However, improved outcomes and earlier weaning of extremely preterm infants might allow earlier body composition assessment.

#### 4.2.2. Repeated Testing

We demonstrated that the highest number of weekly body composition tests (*n* = 3) before discharge were performed in the extremely preterm group (Figure 1). This observation can be explained by the length of hospital stay of infants born at a younger gestational age [31].

We demonstrated that the applicability and usability of ADP measurements during hospital stays significantly vary among different groups of GA at birth (Figure 1):

Extremely and very preterm infants: Although the first measurements were possible starting at 31 weeks PMA, when infants were not receiving respiratory support, the median time was 36 weeks PMA. Despite this relatively late PMA, the prolonged hospital stay allows repeated body composition tests (mean number of measurements = 3) before discharge in this group. This hints at a large enough window for repeated weekly body composition analysis before discharge, opening the option to plot body composition growth trends and introduce nutritional intervention on the basis of body composition data during the hospital stay.

Moderate and late preterm infants: Body composition measurements can be initiated soon after birth (1 ± 0.7 weeks of life). A mean of two ADP measurements before discharge limits longitudinal assessment during the NICU stay. However, it allows individual adjustment of nutritional management. We suggest initiating nutritional adjustments if necessary and performing follow-up measurements after discharge.

Term infants: Due to the short hospitalization period, repeated ADP testing is limited during normal hospital stays. This does not allow continuous plotting of body composition reference charts during hospital stays. Hence, the value of routine body composition assessments in this GA group regarding nutritional monitoring and intervention during hospital stays is limited. Body composition assessments for the adjustment of discharge nutritional regimens, however, could provide value.

#### 4.2.3. Frequency

A recent study by Lücke et al. revealed that at weekly intervals, the reproducibility of the ADP method is sufficient for monitoring body composition along trajectories [24].

At MetroHealth Medical Center, infants are tested once during their hospital stay (either at term or prior to discharge) [19]. In contrast, both the NICU Nuremberg and Cincinnati Children’s Medical Center perform weekly ADP testing.

The decision between repeated or single body composition assessments depends on institutional focus. While single body composition assessments may reduce the workload, continuous body composition assessments provide options for continuous monitoring of nutritional status and for creating body composition growth trajectories. The lack of established recommendations for nutritional or therapeutic consequences from repeated body composition assessments limits the indication for high-frequency testing on a routine basis.

#### 4.2.4. Time and Personnel Requirements

For safety reasons, each ADP test requires at least one PEAPOD operating nurse and one PEAPOD nurse. In our study, the time per ADP test decreased significantly when three, not two, nurses were available (from 13 min to 8 min). This significant reduction in the total time requirement per test was due to an improved, more efficient workflow. Staffing with two PEAPOD nurses will allow the preceding infant to be prepared for the assessment while another infant is still inside the PEAPOD. The time required per test at NICU Nuremberg was slightly greater (8 ± 0.6 min with two PEAPOD nurses) than the 5–7 min testing time estimated by Alja’nini et al. [19]. In his publication, however, it is unclear whether testing cycles included dressing, undressing, and logistics between the unit and the PEAPOD room. Additionally, it remains unclear how much personnel were involved in the presented testing routine, making a comparison of the cumulative work time impossible. For an average of 12 body composition assessments per week, the required cumulative work time at NICU Nuremberg was up to 5 h for all three operators. Overall, the workload is reasonable, and the time-per-test efficiency is optimal for all three operators.

#### 4.2.5. Clinical Significance

Overall, routine body composition measurements provide valuable information for monitoring growth and nutritional status. In particular, high fat mass and reduced fat-free mass are related to suboptimal neurodevelopment and risk for metabolic and cardiovascular diseases later in life [6,32,33]. An optimum body composition is associated with improved neurological outcomes [6,7].

We aim to use body composition data for growth interpretation and to adjust nutritional management by plotting body composition data in reference graphs. These practices, however, are not yet standardized, leaving space for both different interpretations and different interventions (e.g., nutritional management) between medical practitioners and healthcare institutions. Salas et al. reported that simply providing body composition data have little effect on physician decisions or nutritional management [18].

This indicates the need for standardization of (1) the testing routine, (2) visualization and interpretation of body composition data, and (3) nutritional intervention regimens to efficiently use valuable body composition data.

In our NICU, we have taken first steps toward this process by defining an easily reproducible clinical procedure and standardizing the visualization of body composition data via reference charts by Norris et al., Hamatschek et al., and Demerath et al. [34,35,36]. At our hospital, the data from weekly body composition assessments are graphed into reference charts and provided to physicians to allow individual nutritional management. The evaluation and interpretation of individual body composition growth trajectories are performed weekly on the basis of alignment or deviation from percentiles.

This information is obtained by individually adjusting the nutritional supply during hospital stays and modifying postdischarge nutritional regimens on the basis of individual requirements (personalized or precision medicine).

### 4.3. Future Clinical Utility of Body Composition Data

A standardized protocol for routine testing would lower the threshold for implementing measurements in clinical practice while enhancing the comparability of body composition data across different childcare institutions.

Further investigations should address a model on how body composition data can help to further standardize individualized nutritional management by providing different quantities of macronutrients on the basis of infants’ needs (e.g., different protein–energy ratios or carbohydrate–nonprotein ratios).

Therefore, as an important next step, research should aim to determine the optimum body composition that leads to optimum neurocognitive development while preventing vascular and metabolic diseases. Therefore, exact nutritional intervention guidelines must be formulated to guide the interpretation of body composition results in routine clinical practice.

Furthermore, individual nutritional intervention could allow earlier discharge from the hospital.

### 4.4. Limitations of the ADP Method

Noninvasive procedures can be implemented in clinical practice with a reasonable workload. Like all indirect body composition assessments, the PEAPOD device relies on assumptions. The FM density is constant, and the FFM density is only modified by sex and age [26]. Individual changes in body compartment density may lead to errors in estimation. A detailed description of the methodical limitations can be found in a recent article by our group [24]. Nevertheless, the PEAPOD has been validated by various comparability studies [13,14,16,17]. Furthermore, the ADP method was identified as the single “most accurate and reliable method for assessing body composition in preterm infants” in a review analyzing body composition methods in premature infants by Nagel et al. [12].

Another limitation of this method is that infants must be clinically stable before tests can be performed. Hence, body composition tests were initiated at significantly later postnatal and postmenstrual ages in infants with lower GA. Further noninvasive body composition methods, such as new BIA devices, should be reassessed for accuracy and reliability, as they could complement early body composition measurements with the PEAPOD.

### 4.5. Limitations and Strengths of the Study

This manuscript does not address data from body composition testing. Hence, individual fat and fat-free masses were not analyzed. This has been performed in a previous paper [24]. This manuscript solely analyses the applicability of this method in clinical practice and the presented testing protocol in clinical practice for different groups of premature infants.

A limitation of our protocol is that it does not provide a standardized guide for the interpretation and clinical utility of body composition data. This should be the aim of further investigations to improve outcomes.

A key strength of our study is that the PEAPOD tests were integrated into the clinical routine, and the study was performed under “real-life” conditions. During the entire study period, one single PEAPOD operating nurse was responsible for testing, thereby reducing measurement bias. In addition, only clinically stable infants without respiratory support were tested in routine clinical practice, providing a large, homogeneous cohort of both preterm (*n* = 185) and term infants (*n* = 75) with a total of *n* = 429 measurements.

## 5. Conclusions

The retrospective analysis of our data proved the feasibility of weekly routine ADP assessments in preterm infants. However, the initiation of routine testing in extremely preterm infants starts at a significantly greater postnatal age than in more mature groups. ADP assessments can be safely, easily and reasonably integrated into routine clinical practice. Body composition data may be a valuable tool for providing additional information on nutritional status and infant growth. A standardized routine protocol allowing identical measurement conditions across healthcare institutions and a standardized interpretation tool for age-adapted body composition data, however, will improve the comparability and usability of the method.

## Figures and Tables

**Table 1:** Frequency of routine clinical PEAPOD assessments per gestational age (GA) group. Wk. = weeks, m = male, f = female, PMA = postmenstrual age in weeks, * = statistically significant difference between the GA groups (*p* < 0.05).

Groups per GA	Extremely Preterm; <28 wk.	Very Preterm; 28 to 31 + 6/7 wk.	Moderate and Late Preterm; 32 to 36 + 6/7 wk.	Term Infants; ≥37 wk.	All Subjects
Number of subjects (m/f)	14 (11/3)	28 (19/9)	143 (81/62)	75 (48/27)	260 (159/101)
Total number of tests	42	65	244	78	429
Mean GA (weeks)	26.3	30	34.4	39	33.6
Week of life at first PEAPOD test	10.5 ± 3.2 *	4.4 ± 2.4 *	1 ± 0.7 *	0.7 ± 0.6 *	2.5
PMA at first PEAPOD test	36.6	34.2	35.3	39.9	35.5
Number of tests before discharge	3.1 ± 1.4 *	2.4 ± 1.4	1.7 ± 1.1	1.1 ± 0.2 *	1.65 ± 0.9

## Data Availability

The original contributions presented in the study are included in the article, and further inquiries can be directed to the corresponding author.
